# A Guide to Fluorescent Protein FRET Pairs

**DOI:** 10.3390/s16091488

**Published:** 2016-09-14

**Authors:** Bryce T. Bajar, Emily S. Wang, Shu Zhang, Michael Z. Lin, Jun Chu

**Affiliations:** 1 Medical Scientist Training Program, University of California, Los Angeles, CA 90095, USA; BBajar@mednet.ucla.edu; 2 Harvard College, Cambridge, MA 02138, USA; emilyswang@college.harvard.edu; 3 Institute of Biomedical and Health Engineering, Shenzhen Institutes of Advanced Technology, Chinese Academy of Sciences, Shenzhen 518055, China; shu.zhang@siat.ac.cn; 4 Departments of Bioengineering and Neurobiology, Stanford University, CA 94305, USA; mzlin@stanford.edu

**Keywords:** fluorescence resonance energy transfer (FRET), biosensors, fluorescent proteins

## Abstract

Förster or fluorescence resonance energy transfer (FRET) technology and genetically encoded FRET biosensors provide a powerful tool for visualizing signaling molecules in live cells with high spatiotemporal resolution. Fluorescent proteins (FPs) are most commonly used as both donor and acceptor fluorophores in FRET biosensors, especially since FPs are genetically encodable and live-cell compatible. In this review, we will provide an overview of methods to measure FRET changes in biological contexts, discuss the palette of FP FRET pairs developed and their relative strengths and weaknesses, and note important factors to consider when using FPs for FRET studies.

## 1. Introduction

**Figure 1:**
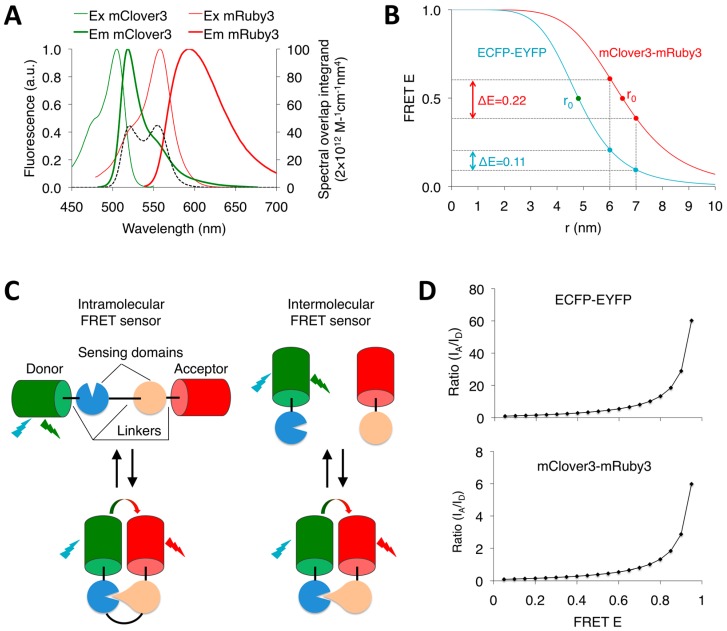
The principle of fluorescence resonance energy transfer (FRET). (**A**) Spectral overlap between mClover3 and mRuby3. The spectral overlap integrand (a product of f_d_(λ), ε_A_ and λ^4^ in the Equation (2)) is indicated by the black dashed line; (**B**) FRET efficiency (FRET E) versus distance. The FRET E varies with the sixth power of distance between donor and acceptor. The Förster radius (r_0_) is the distance at which 50% FRET occurs. Compared to ECFP-EYFP, mClover3-mRuby3 exhibits a larger FRET E change because of the larger r_0_ at which the given FRET biosensor operates; (**C**) Two types of FRET biosensors: intramolecular and intermolecular FRET biosensors. The sensing domains undergo conformational changes (intramolecular) or inter-domain interactions upon biochemical changes, leading to the change in FRET E; (**D**) The relationship between the intensity ratio of acceptor to donor (I_A_/I_D_) and FRET E. The ratio of peaks of the emission spectrum acquired by a sensitivity-normalized spectrum-scanning device is non-linearly related to the actual FRET E. However, it is important to note that ratios taken through filter cubes and cameras are not equivalent to ratios derived from a spectrum-scanning device, as filter cubes pass different amounts of light depending on the transmission spectra and cameras exhibit wavelength-dependent sensitivity.

Förster or fluorescence resonance energy transfer (FRET), first described by Theodor Förster in 1946, is a physical phenomenon in which a donor fluorophore in its excited state non-radiatively transfers its excitation energy to a neighboring acceptor fluorophore, thereby causing the acceptor to emit its characteristic fluorescence [1]. Since FRET is highly sensitive to the distance between donor and acceptor dipoles within the 1–10 nm range, FRET-based biosensors, composed of fluorophores and sensing domains, have been widely adopted as spectroscopic rulers to monitor a variety of biochemical activities that produce changes in molecular proximity, such as protein–protein interactions, conformational changes, intracellular ion concentrations, and enzyme activities [2,3]. An advantage of FRET biosensing over biochemical assays is that it is performed optically, enabling interrogation of live cells in a non-destructive and minimally invasive way [3]. Depending on whether the two fluorophores are conjoined to the same molecule, FRET biosensors can be classified into two categories: (1) intramolecular type, in which donor and acceptor fluorophores are conjoined to the same molecule, whereby conformational changes in the molecule induce FRET changes; and (2) intermolecular type, in which donor and acceptor fluorophores are fused to different molecules, and FRET changes occur when the independent molecules come into close proximity [3] (Figure 1C). In all FRET biosensors, choosing optimal FRET pairs (donor and acceptor fluorophores) are key to the high performance of biosensors in living cells [4].

Three main types of fluorophores have been used as FRET pairs in FRET biosensors: small organic dyes, fluorescent proteins (FPs), and quantum dots (QDs). Unlike dyes and QDs, FPs are genetically encoded and can be particularly useful in live cell FRET imaging. First, FP-based FRET sensors are easily constructed by simply fusing FPs to sensing domains via genetic engineering. In contrast, dyes and QDs do not have the ability to label sensing domains without the aid of antibodies [5], which limits the number of sensors that can be made. Second, FPs confer high cellular specificity by using tissue-specific promoters, and also have high subcellular specificity through introduction of subcellular targeting sequences, which enables FRET probes to report activity solely in cell types of interest or subcellular regions of interest. Third, FP-based FRET sensors can be readily introduced into cells in vitro and in vivo through transfection or virus infection, whereas introducing dye- or QD-based FRET biosensors into cells has been challenging. Fourth, dye- and QD-based sensors are not stable in living cells and can be quickly cleared away from the body in vivo [5]. However, FP-based FRET sensors are stable in cells for long time due to high intracellular stability of FPs. For example, EGFP has a half-life time of greater than 24 h in cells [6]. Lastly, stable cell lines expressing FRET biosensors are easily achievable in the presence of antibiotic pressure, which reduces cell-cell viability in FRET imaging and facilitates high-throughput drug screening [7]. Indeed, the variety of engineered FPs with improved optical properties has made it possible to tailor FPs for FRET pairs with high FRET efficiency and develop highly sensitive FRET biosensors.

In this review, we first discuss the means to measure FRET efficiency and dynamic range. Next, we examine commonly used and recently developed FP FRET pairs and their respective applications, advantages, and disadvantages. Finally, we explore important photophysical considerations when selecting FP FRET pairs, as well as future perspectives on new FP FRET pairs for FRET biosensors.

## 2. FRET Efficiency, FRET Dynamic Range and FRET Measurement

FRET occurs between two fluorophores in close proximity with substantial overlap (>30%) between the donor’s emission and acceptor’s absorption spectra [8] and is characterized by FRET efficiency (E). FRET E refers to the percent of energy transfer from the donor to acceptor fluorophores at a given state and is quantitatively described in the two following equations:

(1) $$E=1/\left(1+r^{6}/{r_{0}}^{6}\right)$$

(2) $$r_{0}=0.02108{\left(\kappa^{2}\phi_{D}n^{-4}\left(\int_{0}^{\infty }f_{D}\left(\lambda \right)\varepsilon_{A}\left(\lambda \right)\lambda^{4}d\lambda \right)\right)}^{1/6}\;(in\;nm)$$

where r is the distance between donor and acceptor dipoles; r_0_ is the distance at which the FRET E is 50%; and κ^2^ is the interdipole orientation factor (assumed to be 2/3 corresponding to a random orientation). Note that the dynamic average assumption that κ^2^ for FPs is 2/3 is not appropriate because autofluorescent FPs do not undergo much rotational diffusion (15–20 ns) during the short excited state lifetime (<5.1 ns) because of their relatively large molecular weights (27 kDa) [9]. However, the exact κ^2^ value for FPs is unknown. Using other values of κ^2^ will change the magnitude of r_0_, but the trends between the different FP pairs will remain the same [10]. Thus far, the Förster radii (r_0_) for FP FRET pairs are based on κ^2^ of 2/3 in almost all FP FRET papers. To make all FP FRET pairs comparable, κ^2^ of 2/3 is still used in this paper just to show the difference of r_0_s from different FP pairs; n is the refractive index of the medium surrounding the fluorophores; φ_D_ is the quantum yield (QY) of the donor in absence of the acceptor; f_D_(λ) is the (wavelength dependent) corrected donor fluorescence intensity at wavelength λ with the total intensity (area under the curve) normalized to unity and is dimensionless; ε_A_ is the (wavelength dependent) extinction coefficient (EC) of the acceptor (in M^−1^·cm^−1^), and λ is the wavelength, whereby the integral term represents the spectral overlap between the donor emission and the acceptor excitation (Figure 1A). Thus, the key elements that determine E of FRET pairs are the spectral overlap, the QY of the donor, the EC of the acceptor, the wavelength, and the interfluorophore distance and orientation. To maximize FRET E, a red-shifted FRET pair with improved optical properties including QY, EC and spectral overlap should be used. For a given FRET pair, the FRET E is proportional to the inverse sixth power of the distance between two fluorophores and works only over a distance shorter than 10 nm (Figure 1B). Given that the chromophores of autofluorescent FPs (directly from amino acids of protein sequence) are centrally buried in the β-barrel structure with a diameter of about 2.4 nm, the effective distance for autofluorescent FP-based FRET pairs is less than 7 nm, resulting in practical maximal FRET efficiencies of 40%–55% [2,4]. By contrast, the non-autofluorescent FP LUMP (lumazine-binding protein) binds noncovalently at its surface to the chromophore molecule ribityl-lumazine, and exhibits a high FRET efficiency of up to 62% when paired with the autofluorescent yellow FP Venus [11].

FRET dynamic range refers to the range of E in which a given reporter operates [2]. Namely, FRET dynamic range can be described with the following equation:

(E_max_ − E_min_)/E_min_ (3)

where E_min_ and E_max_ are the minimum and maximum E of a given FRET biosensor, respectively. FRET dynamic range is essential for detection of cellular events with high sensitivity. Since the FRET E and r are related by a sigmoidal curve with the highest slope at its midpoint (E = 0.5 and r = r_0_), a FRET pair with a r_0_ approximating the r that a given FRET biosensor operates at should be used to maximize FRET dynamic range (Figure 1B). It has been shown that many kinase FRET biosensors operate at a distance far from the r_0_ of CFP-YFP pairs [2]. By using red-shifted FP pairs with a high-QY donor and high-EC acceptor, FRET pairs with larger r_0_s may improve dynamic range in kinase FRET sensors.

In FRET sensors, the FRET change rather than static FRET E is directly correlated with activation or inhibition of intracellular signaling molecules. Thus far, two categories of measurement methods have been developed to measure the FRET change: (1) indirect, which involves measurements of FRET E at different states through spectral imaging FRET (siFRET), acceptor photobleaching FRET (apFRET), and fluorescence lifetime imaging FRET (FLIM-FRET); (2) direct, which directly relates change of fluorescence intensity and polarization to the FRET change. This includes sensitized emission FRET (seFRET) and polarization-resolved FRET (prFRET) (Table 1). Compared to FRET E changes, direct measurements are simple and have high temporal resolution, enabling tracking of fast molecular events and high-throughput drug screening [12].

In spectral imaging-based FRET (siFRET), FRET E is calculated from the Förster equations and from the collected emission spectra of both the donor and the acceptor. In one method, FRET E is determined according to the best fit between a collected emission spectrum of a given FRET biosensor and a theoretical emission spectrum of the corresponding FRET pair calculated from Förster equations [2]. In this method, the FRET distance is also measured. Another method involves acquiring spectral images in which each pixel encodes the composite spectrum from all different fluorescent species, extracting the spectra of the different fluorescent species, calculating the spectral overlap using the Förster Equation (2), followed by solving for r_0_ and E based on the collected emission spectrum [14,15]. Like sensitized emission FRET (discussed below), this method requires photostable donor and acceptor molecules and benefits from acceptors with high quantum yield, which increases sensitized emission. The FRET E change is then calculated by finding the Es at two different states of the biosensor of interest. A drawback of this method, however, is that it can only be used for intramolecular biosensors because it requires a known donor–acceptor ratio.

Acceptor photobleaching FRET (apFRET) is dependent on the energy transfer from the donor to acceptor that quenches donor emission [16]. As the acceptor is photobleached, the donor is de-quenched, such that the complete photobleaching of the acceptor enables the determination of E by indicating what proportion of energy the donor transfers to the receptor, as described by the following equation:

E = 1 − (I_pre_/I_post_) (4)

where I_pre_ and I_post_ are the fluorescent intensities of the donor before and after photobleaching, respectively. apFRET offers a straightforward way to measure FRET efficiency without the need of reference cell measurements, and it is most applicable to fixed cells or tissues, or for live-cell experiments in which unbleached acceptor molecules do not quickly diffuse back into the bleached region [17]. Nonetheless, apFRET is an irreversible endpoint assay, as photobleaching destroys the biosensor signal and prevents multiple sampling from the same set of sensors, which limits its application in monitoring biomolecule dynamics in living cells. Notably, apFRET method assumes that: (1) Photobleaching of the acceptor destroys not only fluorescence but also absorption. Some acceptor FPs, however, can be converted to dark states with red-shifted absorption spectrum, such as red FPs [18], or weakly bright states with blue-shifted absorption, such as YFP [19]. In the former case, acceptor fluorescence is lost while FRET remains, leading to an underestimation of FRET E, whereas an overestimation of FRET E occurs in the latter case due to CFP-like species generated from YFP upon intense light illumination; (2) The molar ratio of fluorescent donor to fluorescent acceptor should be ≤1 (acceptors mature faster than donors). Otherwise, the FRET efficiency will be underestimated and is just the apparent FRET efficiency (E_app_), which is the product of the specific FRET efficiency of the fluorescent donor–acceptor complex and the degree of the complex formation with respect to fluorescent donor [20].

In FLIM-FRET, the nanosecond-scale decay pattern of emission, known as fluorescence lifetime, is measured. Fluorescence lifetime values are measured by exciting the donor with an ultrashort pulse of light and then measuring the photon distribution at the nanosecond scale [21]. Quenching of donor emission by FRET interaction decreases the lifetime, and thus measurement of FRET E is possible by comparing fluorescence lifetimes of the donor in the presence and absence of the acceptor, which is described by the following equation:

E = 1 − (τ_DA_/τ_D_) (5)

where τ_DA_ and τ_D_ are the lifetimes of the donor in the FRET biosensor and the donor alone, respectively. FLIM-FRET is a very robust method because variations in excitation intensity, inner filtering, moderate donor photobleaching and detector sensitivity do not influence fluorescence lifetime [22]. FLIM-FRET has four advantages over intensity-based FRET approaches. First, the fluorescence lifetime decay is not as sensitive to fluorescence intensity, which allows for live cell imaging with less photostable FPs and small animal imaging with high tissue scattering [23,24]. In addition, FLIM-FRET does not require calibration, which should be done in intensity-based FRET using the same detector due to spectral sensitivity issues. Second, the spectral cross-talk and direct acceptor excitation are not big issues because only donor fluorescence lifetime is measured in FLIM-FRET imaging [4]. Third, FLIM-FRET is internally calibrated and therefore independent of donor and acceptor concentrations, the proportion of biosensors that respond, or diffusion that can affect ratiometric signals, which enables the detection of protein–protein interactions in living cells [21]. Fourth, irrespective of single or double exponential decay for donor’s fluorescence lifetime, FLIM-FRET enables the identification of fractions of molecules involved in FRET [23], which allows for true FRET efficiency measurements and quantitative measurements. Since many photons need to be measured to achieve a high signal-to-noise ratio, acquisition times for conventional laser scanning TCSPC FLIM are in the order of minutes [22]. Even with single-photon avalanche photodiodes (SPAD), it acquires a 256 × 256 pixel image with high signal-to-noise ratio in seconds that may impair its use in monitoring fast dynamic processes [13,22]. Additionally, FLIM requires expensive and highly specialized equipment, preventing its wide use in most laboratories.

Sensitized emission FRET (seFRET) is a practical method to measure changes in FRET because of its ease of use and fast imaging times. There are four methods to measure sensitized emission in steady-state images of FRET biosensors: N_FRET_, FRETN, FR, and ratiometric FRET, where FRETN is intensity dependent and not recommended for FRET analysis [25]. N_FRET_ is a method that calculates the FRET change from the intensities of the donor at the donor emission, the acceptor at the acceptor emission, and the acceptor at the donor emission, and corrects for the FRET signals and expression levels of donor and acceptor, which allows N_FRET_ to be especially useful in intermolecular FRET biosensors, where the donor–acceptor ratio is unknown [25]. FR reflects the FRET change as the fractional increase of acceptor emission due to FRET, correcting for spectral crosstalk [26]. Finally, ratiometric FRET is the ratio between the uncorrected FRET signal and the donor intensity at donor excitation. Small changes in FRET are boosted by ratiometric FRET because donor signal correspondingly decreases as FRET signal increases. Ratiometric FRET is most commonly used in intramolecular biosensors because of its simple implementation and lack of correction required for spectral cross-talk. However, it is important to note that ratio change is not equivalent to FRET E change for two reasons. First, the ratio, even if spectral sensitivity of the detection system is uniform across all emission wavelengths as in a well corrected spectrum-scanning device, the ratio is relate to FRET E non-linearly (Figure 1C). Second, ratios of signal intensities acquired with filter cubes and cameras are not equivalent to ratios derived from a spectrum-scanning device, as filter cubes pass different amounts of light depending on the transmission spectra and cameras exhibits wavelength-dependent sensitivity. Thus different microscopes may report different FRET ratio changes. In theory, emission intensities can be adjusted for filter transmission, camera sensitivity, cross-excitation, and bleed-through by taking calibration measurements using each fluorophore alone. However, this is rarely done. This may be because FRET E is not appreciably more useful than simple ratios in relating to a biological parameter, since multiple combinations of interfluorophore distance and orientation can produce the same FRET E, not to mention different degrees of fluorophore maturation.

Fluorescence can be characterized not only by wavelength, intensity, and lifetime, but also by polarization. Upon excitation of the sample with polarized light, only fluorophore molecules with favorable dipole orientation (i.e., parallel) to the excitation light polarization light can be excited. Since autofluorescent FPs are large (27 kDa) and have slow rotational diffusion times of 15–20 ns with short fluorescence lifetime (<5.1 ns), they exhibit very little rotational depolarization and their fluorescence is highly polarized [9,27]. When energy transfer occurs, the fluorescence of acceptor FPs becomes partially depolarized because of different dipole orientation to donor [23]. In polarization-resolved FRET imaging (prFRET), energy transfer can be detected by monitoring changes in polarization through steady-state or time-resolved measurements in the time-domain or frequency-domain and using scanning or wide-field microscopes, in which the intensities of fluorescence polarized parallel and perpendicular to the polarization vector of the polarized excitation source are measured [28]. The prFRET imaging holds a unique advantage over all other FRET approaches in that it is the only technique that can detect homo-FRET: the energy transfer between spectrally identical fluorophores. prFRET has a potentially higher dynamic range and faster detection time than FLIM-FRET [28,29,30]. However, direct excitation of the acceptor and bleed-through of the donor emission in hetero-FRET (spectrally distinct fluorophores) can increase polarization [23]. Therefore, three-filter cube-like corrections are required to eliminate those false positives in hetero-FRET anisotropy [31].

## 3. Types of FP FRET Pairs

### 3.1. CFP-YFP FRET Pairs

**Figure 2:**
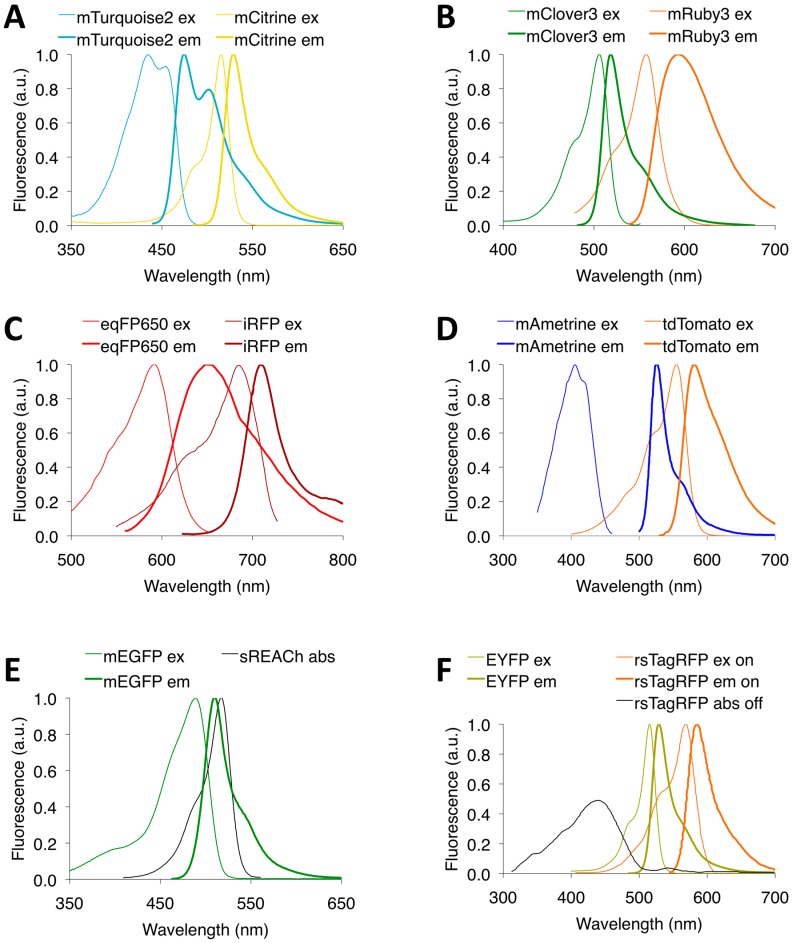
Normalized excitation (or absorbance) and emission spectra of FPs of representative two-color FRET pairs. (**A**) mTurquoise2-mCitrine, a CFP-YFP pair; (**B**) mClover3-mRuby3, a GFP-RFP pair; (**C**) eqFP650-iRFP, an FFP-IFP pair; (**D**) mAmetrine-tdTomato, a LSS-FP based pair; (**E**) mEGFP-sREACh, a dark FP-based pair; (**F**) EYFP-rsTagRFP, an optical highlighter FP-based pair.

The first FP FRET pair developed was composed of enhanced blue FP (EBFP) and enhanced green FP (EGFP), but its low brightness and low photostability made this pair impractical in most applications [4]. Although brighter and more photostable BFPs have since been developed, they still suffer from the phototoxicity of near-UV excitation, limiting their applications in long-term live cell imaging [4]. To overcome the limitations of BFP-GFP pairs, cyan FP-yellow FP (CFP-YFP) pairs were developed and became the most popular FP FRET pairs (Table 2 and Figure 2), starting with the ECFP-EYFP pair [32,33]. One advantage of cyan donors is that a few engineered CFPs have high quantum yield, including mTurquoise2 (QY = 0.93), mCerulean3 (QY = 0.87), mTFP1 (QY = 0.85) and Aquamarine (QY = 0.89) [34,35,36,37], which well-matches their large fluorescence lifetime (Table 2). To our knowledge, mTurquoise2 is the monomeric FP with highest QY so far, providing a large r_0_ of 5.9 nm when paired with sEYFP. Commonly used YFPs include EYFP derivatives mVenus, mCitrine, sEYFP, and YPet, which are less sensitive to pH and chloride, and more photostable with better folding at 37 °C compared to that of EYFP [38,39,40]. However, CFP-YFP pairs suffer from several problems problematic to FRET, including fast photobleaching of YFPs, photoconversion of YFPs into CFP-like FPs, spectral cross-talk, and phototoxicity from violet donor excitation [2]. Furthermore, CFP-YFP-based FRET biosensors exhibit relatively low FRET dynamic range due to low r_0_s (Table 3) when used in kinase FRET biosensors [2]. Besides autofluorescent CFPs, non-autofluorescent FP LUMP (QY = 0.55) was demonstrated to be an efficient FRET donor to Venus [11]. Interestingly, LUMP has unusually long fluorescence lifetime of 13.6 ns, which is largest among all genetically encoded fluorescent protein complexes reported so far (Table 2), leading to high FRET efficiency and dynamic range [11]. However, the ribityl-lumazine molecule is not present in mammalian cells and needs to be exogenously supplied to induce fluorescence. In addition, due to the small size and long fluorescence lifetime of LUMP, LUMP can detect peptide-domain binding with anisotropy measurements [11].

### 3.2. GFP-RFP FRET Pairs

Green-red FRET pairs overcome several disadvantages of cyan-yellow FRET pairs. Excitation of green-red pairs induces less autofluorescence from flavoproteins, less phototoxicity, and greater spectra separation [41]. Until recently, green-red pairs suffered from low brightness of red FPs (Table 2). With the EGFP-mCherry FRET pair, FRET emission is too weak to detect above the donor emission tail, which prevents the use of this pair in ratiometric imaging [42]. However, the EGFP-mCherry pair exhibits decent dynamic range in FLIM-FRET imaging due to the relatively high fluorescence lifetime of EGFP (2.4 ns) and relatively large spectral overlap. To further increase the FRET dynamic range of GFP-RFP pairs in FLIM-FRET imaging, a new green FP NowGFP with a lifetime of 5.1 ns was developed and has been shown to be an efficient donor for mRuby2 [43,44]. The development of new bright and photostable green FP donors and red FP acceptors has improved intensity-based FRET applications (Table 2 and Figure 2). Bright green FP donors include Clover and mClover3, derived from GFP, and mNeonGreen, derived from *Branchiostoma lanceolatum* YFP [45,46]. Bright and photostable red FP FRET acceptors include mRuby derivatives mRuby2 and mRuby3, the latter of which is the brightest and most photostable monomeric RFP yet described [2,45]. The Clover-mRuby2 FRET pair exhibited improved dynamic range compared to CFP-YFP pairs and improved FRET E compared to EGFP-mCherry [2]. Further, mClover3-mRuby3 and mNeonGreen-mRuby3 both have the highest r_0_ (6.5 nm) of any FRET pairs with monomeric FPs to date (Table 3). In sum, bright and photostable green donors and red acceptors have made GFP-RFP FRET increasingly attractive in living cells due to its advantages over CFP-YFP FRET.

### 3.3. FFP-IFP FRET Pairs

In order to monitor molecular processes in most mammalian tissues, FRET pairs with more red-shifted spectra than GFP-RFP are required. Due to their low light scattering and absorbance from hemoglobin, far-red FPs (FFPs) and infrared fluorescent proteins (IFPs) have been particularly useful for deep-tissue imaging [47,48]. Given the large spectral overlap between FFPs and IFPs, FRET can occur in FFP-IFP pairs (Figure 2). The first monomeric IFP, IFP1.4, from the Deinococcus radiodurans bacteriophytochrome, utilizes Biliverdin IXα (BV), which is ubiquitous in mammalian cells, as a chromophore to produce fluorescence [49]. IFP1.4 can be used as a FRET acceptor to the far-red FP mPlum [50], where an mPlum-IFP1.4 tandem was applied to image in both cell culture and xenograft tumors [51]. Another IFP, iRFP, a dimeric IFP engineered from the Rhodopseudomonas palustris bacteriophytochrome, does not require the addition of exogenous BV to boost fluorescence signal and showed a ten-fold brightness improvement over IFP1.4 in live cells, due to its high affinity to BV and cellular stability [52]. When iRFP was tested as an acceptor to different far-red FPs, eqFP650-iRFP showed the greatest dynamic range in a caspase-3 sensor [53] (Figure 2); however, its dynamic range was still significantly lower than those of the best FRET sensors because of the low QY (<0.2) of FFPs. However, FFP-IFP pairs exhibit r_0_s comparable to the best CFP-YFP pairs due to their red-shifted spectra (Table 3). Further development of FFPs with high QY is essential for in vivo imaging studies.

### 3.4. LSS FP-Based FRET Pairs

Another class of FRET pairs includes ones with large Stokes shift (LSS) FPs. LSS FPs can reduce the spectral crosstalk between the donor and acceptor FPs to provide a larger FRET change in ratiometric FRET imaging (Figure 2), and are useful for monitoring multiple processes in a single cell in multicolor FRET imaging. LSS YFP mAmetrine, with violet excitation and yellow emission, exhibits very large r_0_ of 6.6 nm when paired with tdTomato (Table 3) and has been useful for multicolor FRET [61]. However, mAmetrine has poor photostability with half time of 2.8 min (Table 2), which limits its applications in time-lapse imaging. An orange LSS FP LSSmOrange conferred a five-fold brightness improvement over the previous brightest red LSS FPs [62], and was an effective FRET donor to far-red FPs, such as mKate2 [76]. LSSmOrange has excitation at 437 nm and emission at 572 nm, which fills the spectral gap between yellow and green FPs, and red LSS FPs.

### 3.5. Dark FP-Based FRET Pairs

Dark FPs, which have high absorption and very low quantum yield (<0.1), are valuable FRET acceptors for FLIM, as dark FPs are non-fluorescent but retain their absorption properties to enable FRET, thereby enhancing FRET sensing in FLIM-FRET. The intrinsic advantages of the darkness of dark FPs are that: (1) Diminished bleed-through from the acceptor into the donor emission channel allows for accurate measurement of the donor fluorescence lifetime, thereby improving FRET dynamic range; (2) Dark FP acceptors can decrease the possibility of phototoxicity by lowering the excitation intensity and using wider optical filters; (3) Since FRET pairs that contain dark FPs occupy only a small portion of the wavelength spectrum, more FPs of different colors can be used simultaneously for dual-color imaging in FLIM.

Initially, two dark YFP mutants, collectively called REACh (resonance energy-accepting chromoprotein), were engineered from a YFP with low quantum yield, and served as FRET acceptors to EGFP [65]. To improve on REACh, sREACh (super REACh) was engineered with improved maturation, which increased FRET and reduced cell-to-cell variability [77]. One of sREACh’s limitations was the weak fluorescence it produced that led to unexpected artifacts. In 2015, dark GFP ShadowG was engineered from sREACh to have a major reduction in quantum yield [64]. ShadowG has a quantum yield 10-fold lower than that of sREACh2, showed better folding and maturation compared to sREACh2, and served as a robust FLIM-FRET acceptor to EGFP (Figure 2). EGFP-ShadowG reduced spectral contamination and provided more stable, precise, and sensitive measurements in voltage, calcium, and Ras sensors when compared to mCherry and sREACh [64].

### 3.6. Optical Highlighter FP-Based FRET Pairs

Optical highlighter FPs, also known as phototransformable FPs (ptFPs), can undergo light-induced photoactivation, photoconversion, and photoswitching [78]. Optical highlighter FP-based FRET pairs are particularly advantageous over standard FPs in FRET because they provide spectral change on the same samples without the need for corrections based on reference images of control cells. Furthermore, ptFP-based FRET pairs do not rely on the photo-destructive procedures required in apFRET and can provide more information on protein dynamics, such as the mobility of proteins [79], when used in protein–protein interaction studies.

One category of ptFPs includes photoactivatable FPs (PA-FPs), which can be irreversibly activated from a dark state to bright fluorescence emission, and are useful in photoquenching FRET (pqFRET). In pqFRET, a photoactivatable acceptor quenches the donor FP’s emission upon illumination with UV or violet light, which resembles a reverse apFRET [79]. An example is photoactivatable GFP (PA-GFP) [67], which becomes bright when illuminated with 400 nm light. When used as an acceptor to CFP, the photoactivation of PA-GFP absorbed CFP’s emission to gradually quench CFP’s signal [79], thus allowing for FRET measurement that does not require correction for spectral bleed-through.

ptFPs also include photoswitchable proteins, which can reversibly switch back and forth between two absorbing states upon illumination at different wavelengths, and can be used in photochromic FRET (pcFRET). In pcFRET, a photoswitchable acceptor is excited and reversibly alters its absorbance spectrum, which changes the donor–acceptor spectral overlap. The first red photoswitchable FP was rsTagRFP, which switches from fluorescent red to non-fluorescent upon illumination by yellow light, and reverses the process upon blue-light illumination [66]. With EYFP as a donor, EYFP-rsTagRFP was the first FRET pair to be used for pcFRET (Figure 2), as previously, only chemical dyes were used. In 2013, a photoswitchable green non-fluorescent protein named Phanta [68] was developed. Phanta shifts its absorption from 505 nm to 309 nm under the presence of strong cyan light, behaving similarly to the bright green negative photoswitching FP Dronpa [80]. Furthermore, Phanta reliably photoswitches, maintains its absorbance over at least 18 cycles of photoswitching, and is pH-stable. Phanta performed well as an acceptor to EGFP for pcFRET (Table 3). For rsTagRFP, a major drawback is that no other probes with red emission can be used due to overlapping emissions, but Phanta is non-fluorescent, which allows it to be used in conjunction with other probes simultaneously [68].

### 3.7. Multicolor FRET Pairs

The development of new FPs across the spectrum has created a wide array of FRET pair groups suitable for multicolor FRET, which enables the near-simultaneous or simultaneous imaging of different cellular processes in the same cells with more than two FRET pairs. Based on the number of FRET pairs and excitations, multicolor FRET can be divided into three categories: two FRET pairs with two excitations, two FRET pairs with a single excitation, and three FRET pairs.

**Figure 3:**
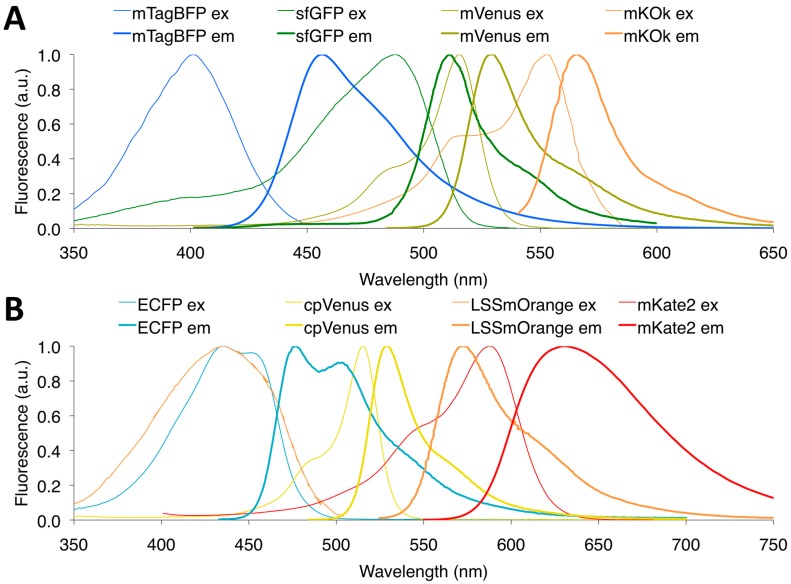
Normalized excitation (or absorbance) and emission spectra of FPs of representative four-color FRET pairs: (**A**) mTagRFP-sfGFP and mVenus-mKOκ pairs, two FRET pairs with two excitations; and (**B**) ECFP-cpVenus and LSSmOrange-mKate2 pairs, two FRET pairs with single excitation.

DsRed, a tetrameric red FP from *Discosoma*, has great spectral overlap with CFP and YFP, which enables it to be an effective acceptor to CFP and YFP [81]. In 2005, using CFP-DsRed and YFP-DsRed, Kawai et al. reported near-simultaneous FRET imaging of initiator- and effector-caspases in the same cell [81]. However, FRET between CFP and YFP may also occur due to heterodimerization of CFP-DsRed and YFP-DsRed via DsRed tetramerization, which could underestimate the FRET dynamic range of CFP-DsRed. To eliminate the dimerization-induced artificial FRET, several multicolor FRET pairs with monomeric FPs were developed. Replacing ECFP and Citrine [43], highly photostable FP mOrange2 was introduced with mCherry into a MT1-MMP sensor. mOrange2-mCherry was then applied with CFP-YFP in the Src sensor to visualize the activities of Src and MT1-MMP almost concurrently [82]. While CFP-YFP and mOrange2-mCherry are spectrally separate, there remains some crosstalk between mOrange2 and mCherry that significantly lowers dynamic range, a problem that may be solved by applying spectrally distinct FPs. In 2008, Grant et al. reported a new method for near simultaneous dual FRET imaging, using TagRFP-mPlum and ECFP-Venus in FLIM [83]. As mPlum is further red-shifted than TagRFP and has the longest emission spectrum, spectral separation could be maximized in this FRET pair combination. Furthermore, the use of FLIM overcomes mPlum’s low quantum yield, as in FLIM, only the donor signal is measured. Finally, in 2013, Su et al. developed a new spectrally separate FRET pair combination for dual imaging, using the FRET pair composed of the blue FP mTagBFP and the green FP sfGFP, with yellow-orange FRET pair mVenus-mKOκ (Figure 3). Using these dual FRET pairs, Src and calcium ion activities were near simultaneously imaged in single living cells without signal distortions from crosstalk [84].

Although two FRET pairs with two excitations perform well in cells, in reality, they cannot visualize two molecular events simultaneously, which complicates the tracking two rapid molecular events, such as calcium and neurotransmitters in neurons. LSS FPs fulfill this need by enabling excitation of spectrally distinct FPs with a single excitation wavelength (Figure 3). In 2008, LSS FP mAmetrine was developed and engineered as a FRET pair with tdTomato, and used to simultaneously ratiometrically image with mTFP1-mCitrine in caspase-3 sensors [61], enabling visualization of different onset times of caspase activity between the nucleus and cytoplasm during apoptosis. In 2009, another LSS GFP, T-Sapphire, was used to develop a FRET pair with RFP dimer2 for single excitation dual FRET imaging. T-Sapphire-dimer2 and ECFP-EYFP were excited with a single excitation of violet light and successfully monitored cAMP and cGMP levels in single cells [85]. Finally, an exceptional FRET donor useful for multicolor imaging is the aforementioned LSSmOrange, an orange FP with a large Stokes shift and a five-fold brightness improvement over the previous brightest red LSS FPs [62]. Using a single-excitation laser, LSSmOrange-mKate2 caspase-3 sensors and CFP-YFP cameleon biosensors were used to accurately and simultaneously image apoptosis and calcium fluctuations, respectively, in real time [62].

Most FRET technologies analyze the interactions between two cellular components. To study more than two protein interactions, three-chromophore fluorescence resonance energy transfer (3-FRET) can measure signals from three mutually-dependent FRET pairs in living cells [86]. While 3-FRET was demonstrated in vitro and in vitro using CFP-YFP, CFP-mRFP, and YFP-mRFP, it was essentially an adaptation of two-color FRET to three possible pairings. To improve upon 3-FRET and provide modeling of FRET efficiencies, three-color spectral FRET microscopy (3sFRET) was developed to study the relationships between three cellular components of interest [87]. In 3sFRET, rather than sequentially applying two-color FRET imaging, a single specimen with three fluorophores fused together is used to analyze a cellular region of interest over time. 3sFRET was validated using mTFP, mVenus, and tdTomato, and then applied to study the interactions between the dimerized transcription factor CCAAT/enhancer binding protein a (C/EBPa) and the heterochromatin protein-1a (HP1a) in live-mouse pituitary cells [87]. In 2013, this technique was extended to N-Way FRET microscopy, an approach where any number of fluorophore interactions can be studied using parallel factor analysis and linear model mixing to determine FRET efficiencies [88].

### 3.8. Homo-FRET Pairs

In addition to FRET between spectrally distinct fluorescent proteins (hetero-FRET), energy can be transferred between proteins tagged with the same fluorophore via homo-FRET. An intrinsic benefit of homo-FRET over hetero-FRET is that protein labeling requires only a single type of fluorophore, and it is particularly useful for investigating homo-oligomerization with high sensitivity because of its ability to detect acceptor-acceptor and donor–donor interactions [23,28]. Moreover, the use of a single fluorophore allows for multi-color prFRET imaging along with spectrally distinct FPs [28]. For homo-FRET, a fluorophore with a small Stokes shift and thus great excitation-emission overlap is necessary [22]. In 2002, anisotropy-FLIM (rFLIM) was developed, where wide-field anisotropy could be measured pixel-by-pixel and was used to detect homo-FRET in EGFP-expressing bacteria [89]. The combination of fluorescence anisotropy and microscopy can be used to study clustering, specifically the distances between fluorophores, number of fluorophores per cluster, and relative orientation of fluorophores [90]. In 2009, to investigate spatial information of clusters, Bader et al. developed a method to quantify protein clustering and determine the average number of fluorophores per cluster per pixel and monomer/oligomer fraction per pixel [90]. This system was used to study the clustering of GPI-anchored proteins using mGFP [90]. In 2012, anisotropy was combined with fluorescence correlation spectroscopy (FCS) in a new single-molecule based method called fluorescence polarization and fluctuation analysis (FPFA). FPFA simultaneously measures homo-FRET, brightness, and correlation time. FPFA was applied using a monomeric version of Venus to measure the number of subunits in the α-isoform of calcium-calmodulin dependent protein kinase-II (CaMKIIα) holoenzyme. In another application, time-resolved fluorescence anisotropy imaging was combined with total internal reflection FLIM to measure homodimerization of the amyloid precursor protein using EGFP [91].

## 4. Considerations When Using FP Pairs

### 4.1. FRET Dynamic Range and FRET Change

In addition to engineering FPs with improved acceptor extinction and donor quantum yields, one strategy to increase FRET dynamic range and FRET change is to engineer FRET acceptors with improved maturation and folding, which is a property of FPs that is often neglected. Folding is not a problem in the case of qualitative studies, such as protein subcellular localization in cells, and quantitative experiments with short imaging windows (minute timescale). However, folding could be problematic if quantitative fluorescence measurements over a long time (tens of minutes to hours) are required and FPs with poor folding are used. Folding could be worse in FRET sensors than in free FPs themselves because FRET biosensors consist of multiple domains. In fact, studies show that while photophysical parameters are good starting points for finding optimal FRET pairs, maturation greatly impacts the resilience of FRET signals, and experimental validation of FPs is essential. Even with worse photophysical characteristics, FPs with faster maturation rates tend to show better FRET performance [10]. For example, mCherry, while having suboptimal brightness properties compared to mRuby2, was a faster folder and thus achieved higher apparent FRET efficiencies [10]. mRuby3, a fast-folding variant of mRuby2, exhibits larger FRET changes [45]. The dark YFP sREACh contains two mutations used in mVenus and mCitrine that improved folding efficiency by 50% and improved signal-to-noise ratio when acting as an acceptor to mEGFP by around 50% compared to its predecessor REACh [77]. As expected, increasing the maturation of REACh also decreased the variability of measured FRET signals [77]. It is essential that the two FPs in a given FRET pair have both equal and fast maturation rates; different maturation rates will change the ratio of acceptor to donor from the theoretical 1:1 ratio, and FPs with slow maturation could mature during imaging and impact FRET quantitative analysis. Good folding is also important to improve FRET dynamic range by enhancing EC and QY, for example SEYFP versus EYFP [55,56].

Moreover, implementing self-associating “sticky” FPs in FRET sensors is another way to improve dynamic range. “Sticky” donor and acceptor FPs contain hydrophobic mutations at the FP dimerization interface, which allow them to form a weak intramolecular complex that strengthens FRET in the high-FRET state and enables dissociation from one another in the low-FRET state. CyPet and YPet were the first reported FPs to have increased FRET dynamic range compared to ECFP and EFYP upon introduction of weak dimerization mutations [92], and the importance of these mutations was further validated through the engineering of “sticky” EYFP and ECFP with two mutations found in CyPet and YPet, which showed a 16-fold emission ratio change in a protease sensor [92]. Similarly, “sticky” variants of red FPs mOrange and mCherry were engineered and used to generate strong FRET sensor responses for protease activity, Zn^2+^, and bile acids [93]. In general, constructing FRET sensors with large dynamic range is traditionally a trial-and-error process because it is difficult to predict the orientation of the FPs, but the use of “sticky” FPs could reduce the initial testing required for optimizing the sensor response [94].

Similar to “sticky” FPs, designing weak helper interactions between FPs can also increase FRET dynamic range by bringing the donor and acceptor into close proximity. In 2013, Grunberg et al. developed two approaches to use synthetic physical “helper” interactions to improve FRET in helper-interaction FRET (hiFRET) pairs [95]. In the first approach, computational methods were used to create an electrostatic interaction between Citrine and mCherry, two FPs originating from two different species, with no intrinsic interactions, while “sticky” FPs are from the same species. With the electrostatic encounter-complex strategy, FRET efficiencies increased around 21.9% without background signals. This method, however, was not applicable to less-controlled environments and cells, so a second approach was developed, where weak WW or SH3 domain-peptide interaction modules were attached to FP pairs. By fusing the WW or SH3 domains and matching peptides to mCitrine and mCherry’s C termini, respectively, FRET efficiencies doubled. Furthermore, mTFP1-mCherry’s FRET efficiency in FLIM also doubled from 15% to 31% when enhanced with WW helper-interactions. Therefore, hiFRET probes may be useful to improve FRET signals, especially as peptide-domain modules can be easily fused to any FP pairs.

Finally, modification of the polypeptide linkers between the FPs and the sensing domains can improve FRET dynamic range. Different linker sequences can shift the distance and orientation of FPs, which result in changes in FRET efficiencies [96]. Current ways to select for optimal linkers include systematically screening through trial-and-error, as single amino acid substitutions alone change FRET drastically [97], or engineering circularly permuted FPs [98]. These approaches, however, are time-consuming, so a rational design approach using molecular dynamics simulations and linker structure prediction would be particularly advantageous. Using this design approach, a new CFP-YFP cAMP sensor improved FRET efficiency by two-fold [99] through the substitution of a more rigid linker, which improved dynamic range as well. In 2011, a modular linker ER/K was engineered that minimizes baseline FRET to increase dynamic range due to its extended alpha helix structure [100]. In another study, dynamic range was improved for intramolecular FRET sensors through using an optimized backbone Eevee [101], which contains a long and flexible linker that renders the sensor “distance-dependent” rather than “orientation-dependent”, as it is difficult to predict and control orientation. When applying Eevee using CFP-YPet FP pairs as the FRET pairs, new FRET sensors for PKA, ERK, JNK, EGFR/Abl, Ras, and Rac1 were generated without additional optimization and all showed increased dynamic range.

### 4.2. Delayed or Decreased on/off Kinetics

One major weakness found in several intramolecular kinase sensors is delayed kinetics. First, the relatively large size of FPs (~27 kDa) slows movement, delaying response time as the FPs must travel in space between states [4]. Second, tight binding of sensing domains may slow off kinetics. For example, in intramolecular kinase sensors, FRET is achieved by the binding of a phosphorylated peptide to a phospho-binding domain, which brings donor and acceptor FPs into proximity. Due to the tight binding of these regions, and possibly due to steric hindrance caused by the conformational change of the reporter itself, the phosphate is not easily accessible by phosphatases, which decreases the off-kinetics of the reporter. One approach to accelerating off-kinetics is to decrease the affinity of the sensing domain: notably, mutations to the calcium-binding site of the calcium sensor TN-XXL increased K_d_ while increasing the off-kinetics of the reporter [102]. Some non-FRET fluorescent reporters have overcome this difficulty by reporting kinase activity with nucleo-cytoplasmic shuttling events [103,104], but FRET remains preferable in terms of providing spatial specificity in signal. If fast kinetics on the order of milliseconds with intracellular specificity are required, sensors based on a single circularly permuted FP (cpFP) would be ideal, such as the calcium indicator GCaMP6f [105] and the voltage sensor ASAP1 [106]. However, few cpFP sensors currently exist due to their difficulty in engineering relative to FRET sensors.

### 4.3. Photostability and pH Sensitivity

The main limitation in long time-lapses in FRET imaging is the photostability of the fluorophores, which will decrease signal over time and affect donor–acceptor ratios in ratiometric FRET. It is possible to calculate photobleaching-corrected FRET efficiency in time lapse imaging through E-FRET [20], a nondestructive FRET imaging method that corrects for intensity loss due to photobleaching, but this method is still temporally limited by the photostability of the acceptor and the signal-to-noise ratio of the fluorophores. Thus, it is most advantageous to use bright and photostable fluorophores that can continue to produce high signal even with long periods of imaging or high intensity excitation. Engineering bright FPs with high photostability has been challenging due to the lack of rational methods for predicting the effects of specific mutations on photostability, although limiting oxygen access to the FP’s chromophore has been proposed as a mechanism for improving photostability [45,63]. A combination of random and site-directed mutagenesis was used to develop bright and photostable FPs, mClover3 and mRuby3 [45]. Thus far, the mClover3-mRuby3 or mNeongreen-mRuby3 pair would be the best choice when used in cultured cells in terms of photostability, brightness and FRET dynamic range, except in apFRET, where FPs with poor stability is preferred.

The environmental pH is another important consideration when performing FRET imaging. In particular, both the lifetime and ratiometric signals of CFP-YFP pairs are affected by pH, which can compromise the fidelity of the reporter signal when used in environments of varying pH [107]. For example, in the AKAR biosensor for protein kinase A (PKA) activity, changing the pH from 7.5 to 5 decreases the FRET ratio by about 40%, whereas the normal FRET change from kinase activity typically results in a 10%–20% ratio change [107]. EYFP and Citrine appear to be strongly pH-sensitive FRET acceptors, and pairing either of these YFP acceptors with a pH-insensitive donor like Aquamarine will not significantly decrease perturbations to lifetime or ratiometric signals [107]. One application of pH-sensitive FPs for example EYFP is to make pH sensors [108].

### 4.4. Oligomerization

FPs that are not completely monomerized by site-directed mutagenesis can form homo-oligomer aggregates at high concentrations or when present in a confined region, such as the plasma membrane. FP aggregation may interfere with the cellular localization or function of the proteins to which they are fused by forming dimerization artifacts [3]. Therefore, use of monomeric FPs in FRET pairs disrupts the interaction between reporter molecules and maximizes signal fidelity. EGFP derivatives have been successfully monomerized with the mutations F223R, L221K, and A206K on the dimerization interface [109], all of which are outer barrel mutations that replace non-polar amino acids with hydrophilic alternatives. However, due to the oligomeric nature of RFPs including orange, red and far-red FPs, many reported monomeric RFPs can dimerize at high concentrations. Thus far, only a few truly monomeric RFPs are reported: mFruits from DsRed [110], mRuby derivatives from eqFP611 [111], and FusionRed from eqFP578 [112]. One way to minimize the dimerization effect is to make tandem FPs, such as tdTomato, which consists of two copies of a dimeric FP; however, this doubles the size of the FP, which may increase interference with the reporter [40].

## 5. Conclusions and Outlook

FP-based FRET sensors have succeeded in exploring the molecular mechanisms underlying cancer, immunological and neurological diseases [113,114] and play a key role in drug discovery [115,116]. Nonetheless, there remain FRET sensors that suffer from low dynamic range when detecting subtle or transient biochemical responses in living cells. For example, RhoA activation in neuronal growth cones during ephrinA-stimulated retraction exhibits only a 5% FRET change with Clover-mRuby2 [2]. Optimizing existing FRET pairs, whether in brightness and folding or maturation, could increase FRET dynamic range.

Currently, mNeonGreen-mRuby3 or mClover3-mRuby3 has the largest r_0_s among all monomeric FP-based FRET pairs and exhibits the largest FRET dynamic ranges in kinase FRET sensors [45]. Compared to mClover3, mNeonGreen is slightly brighter than mClover3 (6%) and more photostable and thus may be a probable starting point for evolving a single bright pair with even larger FRET dynamic range for live cell imaging. The QY of LanYFP, the parental tetrameric FP of mNeonGreen, is 0.95 [46]. Moreover, the highest reported QY of engineered avGFP derivatives is 0.93, from mTurquoise2, while the QY of mNeonGreen is 0.8. This may suggest that variants of mNeonGreen can be developed with even higher QY. Unlike avGFP-derived FPs, many engineered RFPs, even for oligomeric ones, fold poorly or mature slowly in cells [112,117]. Although mRuby3 is better than mRuby2 in maturation or folding, its maturation and folding is still worse than mCherry (unpublished data). In turn, the folding and maturation of mRuby3 can be further improved, which would enable more fluorescent mRuby3 molecules in the FRET complex and result in increased dynamic range. This is especially important when performing long-term imaging, since a FP’s maturation during imaging could induce FRET.

With the development of two-photon FLIM (2pFLIM), FRET imaging has been extended from cultured cells to living animals [118]. Due to the limited number of FLIM-FRET pairs, however, it has been difficult to simultaneously image two molecular events with 2pFLIM in vivo. Developing new FLIM-FRET pairs compatible with existing pairs is required. Many 2pFLIM sensors use EGFP or mEGFP as donors due to efficient FRET with RFPs and high photostability under 2P excitation, and it would be useful to develop a new FRET pair compatible with GFP-based 2pFLIM-FRET pairs that would enable imaging of multiple biochemical events in the same cell in vivo. For this purpose, a cyan-excitable orange or red FP like CyOFP1 would be a good starting point as a donor, as a single 2P excitation wavelength, for example, 940 nm, could excite both donors [71]. CyOFP1 has an unusually high QY of 0.76 and decays as a single exponential with a long lifetime of 3.66 ns, which makes it attractive as a 2pFLIM-FRET donor, but also has some bleed-through in the emission channel for EGFP and is not fully monomeric. A red-shifted monomeric CyOFP1 paired with a far-red acceptor with high EC, for example mCardinal [47], could function with a GFP-based 2pFLIM-FRET pair to monitor two molecular activities simultaneously in vivo.

Overall, the development of a variety of FPs has made FRET viable in a larger number of contexts. Brighter and more photostable FPs have increased the FRET imaging window, large stokes shift FPs have enabled the monitoring of multiple signals simultaneously, and photoswitchable and photoactivatable FPs have provided greater control over imaging without the use of controls. The choice of FP FRET pairs in biosensors is strongly dependent on the system in which the sensor will be used. Factors such as pH and folding or maturation time can significantly affect the photophysical performance of a given pair [119], such that a FRET pair optimized for a particular sensor or particular system may not perform as well in other contexts. Therefore, validation of FP performance in a variety of contexts (e.g., in vivo and in vitro) combined with the optimization of photophysical properties, is crucial to the development of generalizable FRET pairs for single cell or in vivo imaging.

## Figures and Tables

**Table 1:** Comparison of different FRET measurement methods.

	siFRET	apFRET	FLIM-FRET	seFRET	prFRET
Suitable in live cells	yes	no	yes	yes	yes
Temporal resolution	second	no	second *	millisecond	millisecond
FRET E change	yes	yes	yes	no	no
Fluorescence characteristics	spectrum	intensity	lifetime	intensity	polarization
Intramolecular	yes	yes	yes	yes	yes
Intermolecular	no	no	yes	yes	yes
Control cells	yes	no	yes	yes and no	yes
Homo-FRET	no	no	no	no	yes

* under single-photon avalanche photodiodes (SPAD)-based FLIM-FRET imaging [13].

**Table 2:** List of FPs mentioned in this review.

FPs	λ_ex_^a^	λ_em_^b^	ε ^c^	φ ^d^	BR ^e^	pK_a_^f^	Photo-Stability ^g^ (min)	Lifetime (ns)	Maturation ^h^ (min)	Quaternary Structure	Reference
**CFP and YFPs**											
Aquamarine	430	474	26	0.89	23	3.3	79	4.1	2 times slower than ECFP ^i^	weak dimer ^j^	[35]
ECFP	433	475	33	0.4	13	4.7	64	2.3, 3.0 ^k^	ND	weak dimer ^j^	[40,54]
mTurquoise2	434	474	30	0.93	28	3.1	>64	3.8, 4.0 ^k^	ND	monomer	[34]
mCerulean3	433	475	40	0.8	32	4.7	~35	3.7, 3.8 ^k^	K_fold_ = 1.90 ^l^	monomer	[34,37]
LUMP ^m^	420	470	24	0.55	13	ND	ND	13.6	ND	monomer	[11]
mTFP1	462	492	64	0.85	54	4.3	110	3.2	ND	monomer	[36]
EYFP	513	527	83	0.61	51	6.9	60	2.9	K_fold_ = 0.39 ^l^	weak dimer ^j^	[40,55]
mVenus	515	528	92	0.57	53	6	15	3	K_fold_ = 5.62 ^l^	monomer	[55,56] [55,56,57]
sEYFP	515	528	101	0.56	57	6.9	ND	ND	ND	weak dimer ^j^	[32]
mCitrine	516	529	77	0.76	59	5.7	49	3.61	ND	monomer	[40,58]
YPet	517	530	104	0.77	80	5.6	49	ND	ND	dimer	[40]
**GFPs and RFPs**											
EGFP	488	507	56	0.6	34	6	174	2.4	25	weak dimer ^j^	[40]
NowGFP	494	502	57	0.76	43	6.2	ND	5.1	ND	monomer	[43]
Clover	505	515	111	0.76	84	6.1	50	3	30	weak dimer	[2,46]
mClover3	506	518	109	0.78	85	6.5	80	ND	ND	weak dimer	[45]
mNeonGreen	506	517	116	0.8	93	5.7	158	3	10	monomer	[46]
mRuby2	559	600	113	0.38	43	5.3	123	ND	150	monomer	[2]
mRuby3	558	592	128	0.45	58	4.8	349	ND	<150	monomer	[45]
mCherry	587	610	72	0.22	16	4.5	96	1.46	40	monomer	[40,59]
**FFPs and IFPs**											
mPlum	590	649	41	0.1	4	4.5	53	ND	100	monomer	[40,50]
eqFP650	592	650	65	0.24	16	5.7	30 ^n^	ND	ND	dimer	[60]
mCardinal	604	659	87	0.19	17	5.3	730	ND	27	weak dimer	[47]
IFP1.4 ^m^	684	708	92	0.07	6	4.6	ND	ND	114	dimer	[48]
iRFP ^m^	690	713	105	0.06	6	4	ND	ND	168	dimer	[48]
**LSS FPs and FP acceptors**											
mAmetrine	406	526	45	0.58	26	6	2.8	ND	48	monomer	[61,62]
LSS-mOrange	437	572	52	0.45	23	5.7	~2.8	ND	138	monomer	[62]
tdTomato	554	581	138	0.69	95	4.7	98	3.1	60	pseudo monomer	[40,63]
mKate2	588	633	63	0.4	25	5.4	81	ND	38	weak dimer	[47]
**Dark FPs**											
ShadowG	486	510	89	0.005	0	ND	ND	ND	76	monomer	[64]
REACh1	495	530	ND	ND	NA	ND	ND	ND	ND	weak dimer	[65]
REACh2	510	538	ND	ND	NA	ND	ND	ND	ND	weak dimer ^j^	[65]
sREACh	517	531	115	0.07	8	ND	ND	ND	133	weak dimer ^j^	[64]
**Phototransformable FPs**											
rsTagRFP	440	585	5 ^o^	0.005 ^o^	~0 ^o^	6.6	ND	ND	43	weak dimer	[66]
			15 ^p^	0.001 ^p^	~0 ^p^						
	567	585	37 ^o^	0.11 ^o^	4 ^o^						
			2 ^p^	0.11 ^p^	0.2 ^p^						
PA-GFP	504	517	17	0.79	14	ND	ND	ND	ND	weak dimer ^j^	[67]
Phanta	506	516	98	0.003	0	4.5	ND	ND	ND	monomer	[68]
**FPs for Multicolor FRET**											
T-Sapphire	399	511	44	0.6	26	4.9	25	ND	78	weak dimer	[40,69]
mTagBFP	402	457	52	0.63	33	2.7	ND	2.6	ND	monomer	[70]
sfGFP	485	510	83	0.65	54	5.5	157	ND	ND	weak dimer ^j^	[46]
CyOFP1	497	589	40	0.76	30.4	5.5	111	3.6	15	weak dimer	[71]
mOrange2	549	565	58	0.6	35	6.5	228	ND	270	monomer	[63]
mKOκ	551	563	105	0.61	64	4.2	ND	ND	ND	monomer	[42]
TagRFP	555	584	100	0.48	49	3.8	37	2.3	100	weak dimer	[63,72]
DsRed	556	586	57	0.79	45	16	~678	3.65	~600	tetramer	[58,73,74]

^a^ Excitation maximum in nm; ^b^ Emission maximum in nm; ^c^ Peak extinction coefficient in mM^−1^·cm^−1^; ^d^ Quantum yield; ^e^ Brightness; product of **ε** and φ; ^f^ pH at which the fluorescence intensity is 50% of its maximum value; ^g^ The time to photobleach from 1000 down to 500 emitted photons per second; ^h^ The time for fluorescence to obtain half-maximal value after exposure to oxygen; ^i^ Maturation of Aquamarine is based on comparison of ECFP and Aquamarine and is its better photostability is likely due to increased rigidity; ^j^ Can be made monomeric with A206K mutation; ^k^ Phase/modulation lifetime; ^l^ Refolding rate from denatured protein in 10^−2^/s; ^m^ Non-autofluorescent FPs. Biliverdin IXα (BV) and 6,7-dimethyl-8-(1′-dimethyl-ribityl) lumazine are the chromphores for NIR FPs and LUMP, respectively. ^n^ Photostability of eqFP650 is based on a comparison of photostability between eqFP650 and mCherry; ^o^ ON state of rsTagRFP; ^p^ OFF state of rsTagRFP.

**Table 3:** List of commonly used and large-r_0_ FP-based FRET pairs.

FRET Pair	φ_D_^a^	ε_A_ (mM^−2^·cm^−1^) ^b^	r_0_ (nm) ^c^	Reference
ECFP-EYFP	0.4	83	4.9	[75]
mTurquoise2-sEYFP	0.93	101	5.9	[2]
mTurquoise2-mVenus	0.93	92	5.8	[35]
EGFP-mCherry	0.6	72	5.4	[2]
Clover-mRuby2	0.76	113	6.3	[2]
mClover3-mRuby3	0.78	128	6.5	[45]
mNeonGreen-mRuby3	0.8	128	6.5	[45]
eqFP650-iRFP	0.24	105	5.8	this work ^e^
mAmetrine-tdTomato ^d^	0.58	138	6.6	this work ^e^
LSSmOrange-mKate2 ^d^	0.45	63	7.0	this work ^e^
EGFP-sREACh	0.6	115	5.8	[64]
EGFP-ShadowG	0.6	89	4.7	[64]
EGFP-activated PA-GFP	0.6	17	4.4	this work ^e^
EGFP-Phanta	0.6	98	5.8	this work ^e^
mTagBFP-sfGFP	0.63	83	4.6	this work ^e^
mVenus-mKOκ	0.57	105	6.3	this work ^e^
CyOFP1-mCardinal ^d^	0.76	87	6.9	this work ^e^

^a^ Quantum yield of donor; ^b^ Extinction coefficient of acceptor; ^c^ Calculated Förster radius assuming random interfluorophore orientation (κ^2^ = 2/3); ^d^ Larger r_0_, relative to mClover3-mRuby3, is due to red-shifted spectra; ^e^ Calculated in this work using Equation (2).
